# Discovering candidate SNPs for resilience breeding of red clover

**DOI:** 10.3389/fpls.2022.997860

**Published:** 2022-09-28

**Authors:** Johanna Osterman, Cecilia Hammenhag, Rodomiro Ortiz, Mulatu Geleta

**Affiliations:** Department of Plant Breeding, Swedish University of Agricultural Sciences, Lomma, Sweden

**Keywords:** pool-seq, phylogenetic tree, DAPC, LASSO, bioclimatic variables, red clover

## Abstract

Red clover is a highly valuable crop for the ruminant industry in the temperate regions worldwide. It also provides multiple environmental services, such as contribution to increased soil fertility and reduced soil erosion. This study used 661 single nucleotide polymorphism (SNP) markers via targeted sequencing using seqSNP, to describe genetic diversity and population structure in 382 red clover accessions. The accessions were selected from NordGen representing red clover germplasm from Norway, Sweden, Finland and Denmark as well as from Lantmännen, a Swedish seed company. Each accession was represented by 10 individuals, which was sequenced as a pool. The mean Nei’s standard genetic distance between the accessions and genetic variation within accessions were 0.032 and 0.18, respectively. The majority of the accessions had negative Tajima’s D, suggesting that they contain significant proportions of rare alleles. A pairwise F_ST_ revealed high genetic similarity between the different cultivated types, while the wild populations were divergent. Unlike wild populations, which exhibited genetic differentiation, there was no clear differentiation among all cultivated types. A principal coordinate analysis revealed that the first principal coordinate, distinguished most of the wild populations from the cultivated types, in agreement with the results obtained using a discriminant analysis of principal components and cluster analysis. Accessions of wild populations and landraces collected from southern and central Scandinavia showed a higher genetic similarity to Lantmännen accessios. It is therefore possible to link the diversity of the environments where wild populations were collected to the genetic diversity of the cultivated and wild gene pools. Additionally, least absolute shrinkage and selection operator (LASSO) models revealed associations between variation in temperature and precipitation and SNPs within genes controlling stomatal opening. Temperature was also related to kinase proteins, which are known to regulate plant response to temperature stress. Furthermore, the variation between wild populations and cultivars was correlated with SNPs within genes regulating root development. Overall, this study comprehensively investigated Nordic European red clover germplasm, and the results provide forage breeders with valuable information for further selection and development of red clover cultivars.

## Introduction

Red clover is a perennial forage legume that grows in temperate regions worldwide. Due to its high protein content, it is considered an important crop for the ruminant industry (Smith et al., 1985; Taylor and Quesenberry, 1996a). In addition to its great nutritional value, red clover provides several important ecosystem services. Due to its symbiotic relationship with nitrogen-fixing bacteria (Sturz et al., 1997; Thilakarathna et al., 2017), red clover increases soil fertility. Compared to alfalfa and white clover, which have similar symbiotic relationships with the *Rhizobium* bacteria, red clover is more efficient at nitrogen fixation (Dhamala et al., 2017). As a perennial crop, red clover also contributes to soil carbon sequestration, reduces soil erosion during the winter, and suppresses weeds (McKenna et al., 2018). However, persistence is generally low in red clover, which adversely affects its overall performance as a forage crop (Taylor and Quesenberry, 1996b). Red clover is a diploid species (2*n* = 2*x* = 14); however, tetraploid (2*n* = 4*x* = 28) cultivars have been developed through chromosome doubling techniques (Taylor and Quesenberry, 1996c). The tetraploid red clover cultivars generally have a higher green biomass yield as well as a higher persistence and resilience than the diploids (Öhberg, 2008). However, their seed yield is generally lower than that of the diploids because of their flower anatomy and a higher rate of embryo abortion (Amdahl et al., 2016).

The development of modern DNA marker-based plant breeding techniques for red clover is lagging behind, despite its economic and ecological significance, although it has been picking up pace in recent years. For instance, the publication of its reference genome (De Vega et al., 2015) has facilitated the discovery of quantitative trait loci (QTL) for various traits, and the “mining” of single nucleotide polymorphism (SNP) markers (Herrmann et al., 2008; Ergon et al., 2019; Li et al., 2019). In two recent papers on population genetics, SNPs were used to assess the population structure in individually genotyped red clover (Jones et al., 2020; Osterman et al., 2021). Jones et al. studied 75 accessions from Europe, Asia, and Iberia where 70 were wild populations and five were commercially available breeding populations. They found that the population structure of red clover is highly correlated with its geographical location and associated climatic conditions. Osterman et al. focused more on the genetic differences between accessions representing different populations and found that, for instance, wild populations were clearly differentiated from cultivated populations. Both studies noted the effect of the outcrossing nature of red clover in the overall higher heterozygosity which decreases the levels of genetic differentiation. Since red clover is a strictly outcrossing species, genetic research should ideally be performed at a population level. Currently, it is quite expensive to sequence an adequate number of individuals within each population for a comprehensive genetic analysis of multiple populations. With a method that is generally referred to as Pool-seq (Schlötterer et al., 2014), individuals can be pooled and sequenced simultaneously using different next-generation sequencing (NGS) methods. SeqSNP is a targeted genotype by sequencing method for genotyping known SNPs, which is also amenable to *de novo* discovery of SNPs located close to the target SNP positions (Osterman et al., 2021). Hence, Pool-seq via SeqSNP is an NGS method in which individuals are sampled, pooled, and sequenced together, targeting known SNP loci. The target SNPs can be selected from the available SNP databases or developed through allele-mining approaches based on existing genomic resources.

SNP markers are codominant single nucleotide markers that have been widely used in various applications, including genomic selection (GS; Heffner et al., 2009) and marker-assisted selection (MAS; Lande and Thompson, 1990). Compared to the phenotype-based selection, these two breeding methods are quicker and can facilitate the development of high-yielding cultivars that are resilient and nutritious within a shorter period of time. Gene-specific SNP markers are preferred over SNPs in other genomic regions since they are more likely to be associated with genes that regulate desirable traits (Poczai et al., 2013). Hence, genes that are highly desirable from the viewpoint of plant breeding can be targeted for genetic diversity analyses. This will enable the determination of suitable genetic resources that could be used in plant breeding programs. Because genetic similarity between populations might reflect phenotypic similarity, gene-specific markers could provide crucial insights into the differentiation of populations in terms of traits, such as growth and development.

Due to its proximity to the North Pole and the effects of the passing Gulf Stream, the Nordic Region of Europe has highly variable weather with large differences in day length over seasons despite its geographically small area. Consequently, the region requires unique crop cultivation conditions, and the key to crop persistence could be found in its wild relatives. In this regard, genetic analyses of both wild and cultivated gene pools could link the breeding material used by Scandinavian breeding enterprises to resilient wild populations.

The purpose of this study was to compare and examine the genetic resources of red clover available in northern Europe by targeting its cultivated and wild gene pools that represent the Nordic countries. Here, SeqSNP was used to target SNPs within genes that influence growth and development, as well as disease resistance. Moreover, population genetic analyses were carried out in order to determine the correlation between genetic differences among wild populations and bioclimatic variables at the original collection sites.

## Material and methods

### Selecting germplasm and planting

For this study, 382 accessions of red clover were used that originate from different parts of the Nordic Region of Europe (**Supplementary Table 1**). Of these, 294 accessions were obtained from NordGen (a regional genetic resources center for the Nordic countries) and 88 accessions from Lantmännen Seed (a plant breeding and agricultural seed company based in Sweden). The NordGen accessions were selected based on their passport data to represent a variety of available germplasm (cultivars, breeding populations, landraces, and wild populations) representing most of the Nordic region of Europe (i.e., Sweden, Norway, Finland, and Denmark). One Russian accession was also included as it was located at the Finnish border. The Lantmännen varieties include cultivars and synthetic populations from the Scandinavian forage breeding programs (Lantmännen, Sweden; Graminor, Norway; and DLF, Denmark), and hence reflect the variety of cultivated red clover available in northern Europe. These accessions include both diploid and tetraploid types that are categorized either as late or middle-late based on their maturation period.

The accessions were planted and grown for two weeks in a greenhouse at the Swedish University of Agricultural Sciences (SLU, Alnarp, Sweden), as described in Osterman et al. (2021). The BioArk leaf collection kit (LGC Biosearch Technologies) was used to collect ten 6 mm leaf discs (1 leaf disc/plant). One pool of leaf tissue representing ten plants was sampled for each accession separately. DNA extraction and genotyping were conducted at LGC Biosearch Technologies (Berlin, Germany), as described in Osterman et al. (2021).

### Genotyping and variant calling

For genotyping, SeqSNP was used with a set of 400 target SNPs developed by Osterman et al. (2021). The SNPs were identified from publicly available red clover genomic resources by targeting coding sequences of genes known to be involved in growth and development as well as in the response to biotic and abiotic stresses. In addition to genotyping the target SNPs, SeqSNP was used to discover novel SNPs in the regions surrounding the target SNPs. In this analysis, 2 × 150 bp reads were used as a sequencing mode. The sequencing depth was set to 50 million read pairs (15 GB raw data) per sample to ensure sufficient coverage of each genotype in each pool thus adjusting the sequencing depth to ×501. SNP calling was performed by aligning the quality trimmed reads to the reference genome using Bowtie2 v2.2.3. For variant discovery, Freebayes2 was used with ploidy set to diploid and minor allele frequency set to 1%. To exclude calls due to sequencing error, allele counts below eight were set to zero as per the recommendations of LGC Biosearch Technologies where genotyping was conducted. The allele frequency of each accession at each locus was calculated based on the read counts.

For determining the validity of converting the read counts of pool-seq into allele frequencies for data analysis, five randomly selected accessions were genotyped at both the individual and pool levels. Following this, the read counts of each pooled sample were converted to allele frequencies. A subsequent step involved converting the genotypic data of the five individuals of each accession into allele frequency data for that particular accession. This was followed by correlation analysis between the allele frequencies obtained from individual genotype sequencing and PoolSeq, which revealed a highly significant correlation (r > 0.95; P < 0.001) between them. Hence, SeqSNP is a highly reliable method to generate data for allele frequency-based data analyses.

Among the 400 target SNP loci genotyped, 5.5% were mono-allelic, 86% were bi-allelic, 7.5% were tri-allelic, and 0.75% were tetra-allelic across the 382 accessions studied. The remaining 0.25% were INDELs (insertion or deletion of a nucleotide). The *de novo* SNP and INDEL calling generated 347 SNPs and 16 INDELs. Among the 347 novel SNPs, 91% were bi-allelic, 8% were tri-allelic, and 1% were tetra-allelic. Due to the complexity of analyzing pooled sequencing data and a mixture of diploid and tetraploid accessions, only polymorphic bi-allelic markers were used. Additionally, tetraploids were treated as diploids as described in Osterman et al. (2021). Overall, 344 originally targeted and 317 *de novo* discovered bi-allelic SNPs (661 SNPs in total), all with minor allele frequency of 5% or above, were used for data analyses in this study.

### Genetic parameter estimation

Tajima’s D was estimated using PoPoolation (Kofler et al. 2011b) based on the quality- trimmed reads combined in a sync file using the respective reference sequences to map the reads. The allele counts from Freebayes2 were imported into R (R Core Team, 2013) and the expected heterozygosity for each population (H_S_) was calculated using the adegenet package (Jombart, 2008). Using the poolfstat package (Gautier et al., 2022) in R, pairwise F_ST_ was calculated for each pair of SNPs as well as for each pair of accessions. After grouping the accessions according to their origins or types an additional pairwise F_ST_ analysis was performed. Nei’s standard genetic distance between populations and between groups (as for the F_ST_ analysis) was calculated using adegenet package. Additionally, Mantel’s randomized test comparing Nei’s standard genetic distance with the geographic coordinates of the germplasm collecting sites of the wild populations was performed to determine whether isolation by distance (IBD) has a significant effect on the genetic differences between the accessions.

### Determining population structure via clustering

Both principal component analysis (PCA) and principal coordinate analysis (PCoA) were used to determine the genetic relationships between the accessions. The PCA was conducted using the pcadapt package (Luu et al., 2017), and SNPs that were most associated with the variation described in the first two principal components were extracted for further analysis. The PCoA was performed using the stats package in R (R Core Team, 2013) based on the Nei’s genetic distance. The Nei’s genetic distance based relationship between the accessions was further analyzed using ComplexHeatmaps (Gu et al., 2016), which generates heatmaps. These analyses enabled the comparison of the accessions both individually as well as collectively based on their origins and types.

The Nei’s genetic distance based relationship between the accessions was further investigated through neighbor-joining (NJ) cluster analysis. The NJ tree was built using the ape package (Paradis et al., 2004) and visualized using the ggtree package (Yu et al., 2017). Incorporating bioclimatic variables to the NordGen accessions of wild populations and maturity types to the Lantmännen accessions into the analyses was made possible using the ggtree package. A map of collection coordinates for landraces, some cultivars and breeding populations as well as wild populations provided by NordGen was constructed using the rnaturalearth package, which uses maps from Natural Earth, in R.

A discriminant analysis of principal components (DAPC) was performed using adegenet with the method described by Jombart et al. (2010); Jombart and Collins (2015) on the allele frequencies. The find.clusters function was used to find the most optimal number of clusters based on the BIC score, and the cluster solution with the lowest BIC score was chosen. The xval function with a test set of 90% with 30 repetitions was used to find the appropriate number of principal components (PCs). This resulted in a five-cluster solution involving 150 PCs.

### Environmental data for NJ tree and LASSO models

Bioclimatic variables were retrieved from WorldClim (Fick and Hijmans, 2017) with a spatial resolution of 30 seconds (~ 1 km^2^) and imported via the raster (Hijmans et al., 2012) package in R. The coordinates of the germplasm collecting sites of the wild populations were used to extract environmental data with interpolation, hence minimizing the effect of potential recording errors. The bioclimatic variables were evaluated based on how they vary within Scandinavia. Most of the precipitation variables were similar across the sampling sites, and therefore they were excluded. The final set of bioclimatic variables from WorldClim include annual mean temperature and precipitation, as well as isothermal and precipitation seasonality. Annual snow coverage was estimated using snow thickness data retrieved from Climate Data Store (CDS) for months with snow (September to June) from 1980 to 2000. The mean snow thickness for each month at a sampling site during the years 1980 to 2000 was calculated and plotted with months on the x-axis and mean snow thickness on the y-axis. The area under the curve (AUC) of the yearly trend was calculated using the AUC function from the DescTools package (Andri, 2021) in R. The AUC value was chosen to represent mean snow coverage for each collection site.

### SNPs with significant contribution to the variation explained by PCA

The pcadapt package (Luu et al., 2017) was used to perform a PCA with the Mahalanobis’ method as the function argument. Thus, the Mahalanobis’ distance was used to measure the extent to which each SNP is related to the first, in this case, two PCs. A χ^2^-test was performed on the SNPs Mahalanobis distance to find those SNPs with significant contribution to the population structure, and the Benjamini–Hochberg correction was applied to control false positive discovery. The significant SNPs were then validated for their biological roles using gene ontology (GO) enrichment to find possible molecular functions differentiating the populations in the PCA clusters.

### LASSO models

The least absolute shrinkage and selection operator (LASSO) regression model was used to connect environmental variables to the allele frequencies. As the number of SNPs exceeded the number of populations in this study, LASSO was considered an appropriate model due to the application of penalization and feature reduction. Among the accessions studied, only those representing wild populations were used for the LASSO models. This is because they could be considered to have adapted to the environments of the sampling sites. Considering the number of SNPs available for the data analysis was high (661), a method for selecting only the most relevant ones was devised to increase the biological aspect of model training. The goal of this analysis was to find the SNPs that genetically differentiated two populations. Thus, the F_ST_ values of all 661 SNPs between every pair of populations were calculated and the top 1% values of each pairwise calculation was selected. A final set of 430 SNPs with high F_ST_ values was selected for the LASSO models. The caret package (Kuhn, 2008) in R was used to train and select the LASSO model. A leave one out cross validation (*LOOCV*) approach was used to train a regression model (*glmnet*) and the mean average error (*MAE*) was computed for model selection. The MAE and root mean square error (RMSE) were compared to the variance of each climate variable to validate the final model; and the error was smaller than the standard deviation of the input for all models. To validate the models further, a linear regression analysis was performed on the bioclimatic variables without including the SNP data. In cases where the LASSO model had a lower RMSE than the linear model, the SNPs were considered to have an effect.

### Validating selected SNPs in terms of biological functions

The analysis of SNP effect on population differentiation in the PCA and LASSO models resulted in nine sets of SNPs, two from the PCA and seven from the LASSO models. A gene ontology (GO) enrichment analysis was carried out on each set of SNPs to find any biological function underlying the population differentiation. The GO analysis was performed with the workbench at dicots Plaza 5.0 (Van Bel et al., 2021) where the correct names of the genes containing the SNPs were determined via the integrated BLAST function. Then enrichment was performed using all red clover genes as background with p-values showing significant enrichment adjusted following Bonferroni’s correction.

## Results

The SeqSNP-based sequencing of the 382 red clover accessions resulted in 661 bi-allelic SNP markers, which were then used for population genetics analysis of the accessions (**Supplementary Table S2**; Osterman et al., 2021). Additionally, 49 tri-allelic, four tetra-allelic and 17 INDELs were identified across the 400 target SNP loci, and 357 SNP loci were discovered *de novo*. Of the 317 *de novo* discovered bi-allelic SNPs, 292 were reported in Osterman et al. (2021) whereas the remaining 25 were specific to this study. It is evident from the number of *de novo* SNPs discovered in this study, compared to that of Osterman et al. (2021), the number of accessions studied had an effect on the number of novel SNPs discovered. Only bi-allelic SNPs were used for the data analyses for the sake of simplicity. At each of the 661 bi-allelic SNP loci, the allele frequency was calculated based on the read counts obtained from the sequencing. The read counts across the 661 bi-allelic SNPs ranged from eight to 4320. Although the range of the allele counts is large, there was no need to scale the frequencies since they were calculated independently for each locus of each accession.

### Genetic variation within and among groups

For data analysis, the accessions were grouped based on their origins and population types. The grouping results in nine origin-based groups (Denmark, Finland, Graminor, Lantmännen, Norway, Sweden, DLF, Local population, and Russia) and eight population type-based groups (Breeding population, Cultivar, Diploid, Graminor, Landrace, Tetraploid, Unknown, and Wild Population). Because DLF, Local population, and Russia (among the origin-based groups) and Graminor and Unknown (among the population type-based groups) had only one accession each, they were excluded from some analyses.

**Figure 1:**
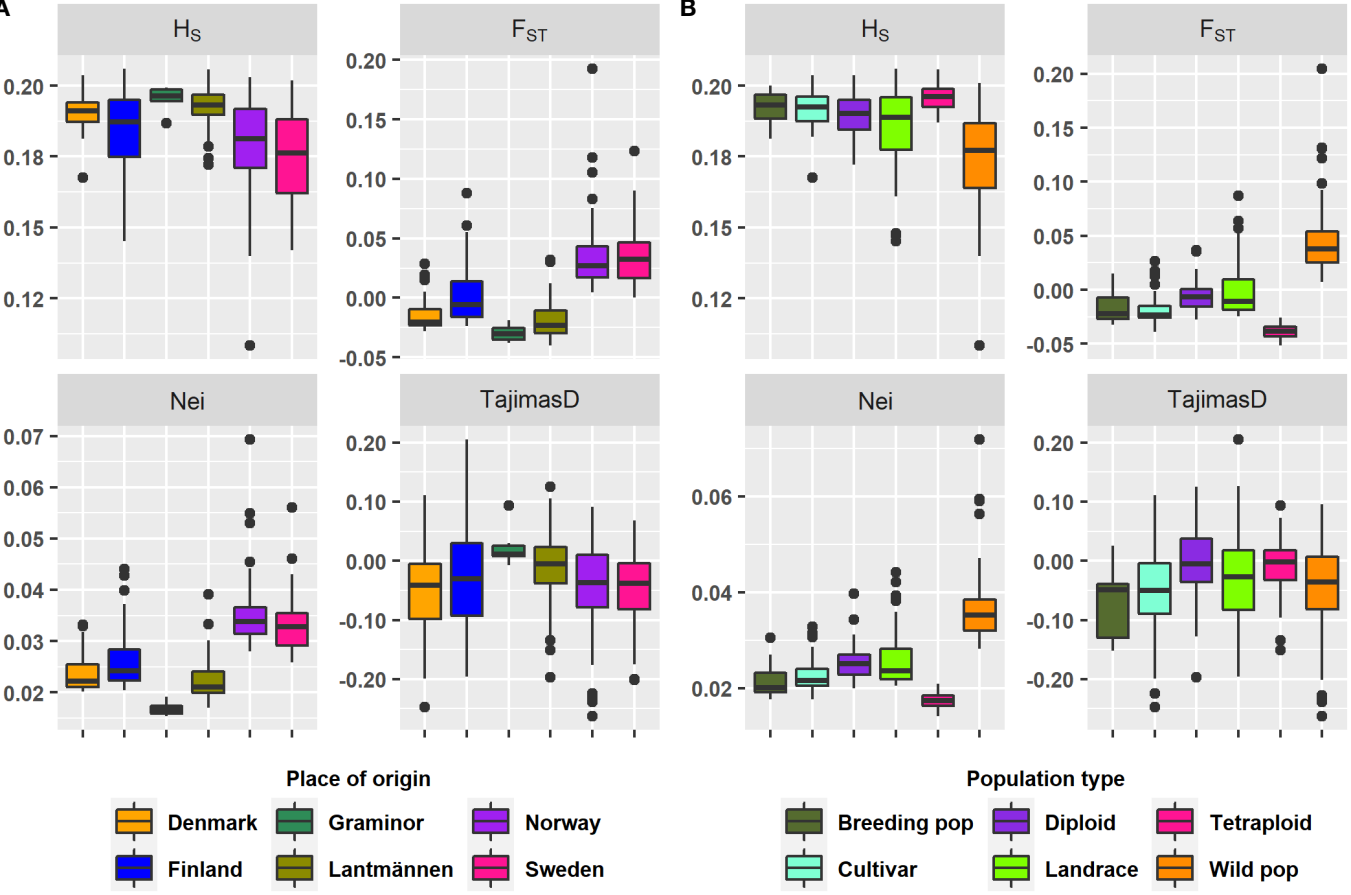
A box plot depicting the range and median for the genetic parameters on each group according to **(A)** Origin and **(B)** Type. The genetic parameters were H_S_, mean expected heterozygosity; Nei, Nei’s standard genetic distance; F_ST_, mean fixation index; Tajima’s D, Tajima’s population genetic test statistic.

The study revealed low genetic diversity and population structure considering the median and mean values of Nei’s standard genetic distance and F_ST_ of each group (**Table 1**, **Figure 1**). Additionally, the results show a difference in the amount of rare alleles present within groups where wild populations had larger levels of rare alleles than cultivated accessions (Tajima’s D in **Table 1** and **Figure 1**). All cultivated groups (breeding populations, cultivars, diploids and tetraploids) had negative F_ST_ mean values. Both negative and zero F_ST_ values indicate lack of genetic variation distinct to each of the populations compared. Only wild populations had a positive mean F_ST_ value, hence, it is the only group (among population types) with any population structure. In the case of origin-based groups, the mean F_ST_ values were negative for Lantmännen, Graminor and Denmark, zero for Finland, and positive for Norway and Sweden. Apparently, the F_ST_ values of the different population type-based groups and origin-based groups were related due to the accessions they shared. The majority of the landrace accessions belong to Finland, and consequently the mean F_ST_ values for both groups were zero. Similarly, most of the accessions from Denmark were cultivars, and hence both Denmark (origin) and cultivars (population type) had a negative mean F_ST_ value. The mean F_ST_ for Sweden and Norway was higher, as they were mainly comprised of wild populations.

**Table 1:** The first column indicates the number of samples in different groups of red clover populations grouped according to their origin or population type.

Grouped by origin	N° samples	H_s_	Nei	F_st_	Tajima’s D	B	C	D	G	L	T	U	W	
**Denmark** ^a^	35	0.19	0.02	-0.01	-0.5	3	86						3	
**Finland** ^a^	72	0.18	0.03	0	-0.03		4			88			15	
**Graminor** ^a^	6	0.19	0.02	-0.03	0.02			33	17	50				
**Lantmännen** ^a^	81	0.19	0.02	-0.02	-0.01			44			56			
**Norway** ^a^	92	0.18	0.03	0.03	-0.04	5	1			4			89	
**Sweden** ^a^	95	0.18	0.03	0.03	-0.05	1	7			9		1	81	
**DLF** ^a^	1	0.20	-	-	-0.02						100			
**Local population** ^a^	1	0.20	–	–	0.06		100							
**Russia** ^a^	1	0.16	-	-	0								100	
**Grouped by type**						**Da**	**F**	**G**	**La**	**N**	**S**	**Df**	**Lo**	**R**
**Breeding population** ^b^	10	0.19	0.02	-0.02	0.07	40				50	10			
**Cultivar** ^b^	41	0.19	0.02	-0.02	-0.05	74	7			2	17			
**Diploid** ^b^	43	0.19	0.03	-0.01	-0.01			5	93				2	
**Graminor** ^b^	1	0.19	–	–	0.01			100						
**Landrace** ^b^	71	0.19	0.03	0	-0.02		81			6	13			
**Tetraploid** ^b^	45	0.20	0.02	-0.04	-0.01	91		7				2		
**Unknown** ^b^	1	0.17	-	-	0.04						100			
**Wild population** ^b^	172	0.18	0.04	0.04	-0.05	0.5	6			48	45			0.5

^a^= a group of accessions belonging to geographic origin; ^b^= a group of accessions belonging to population type; H_s_, mean expected heterozygosity; Nei, Nei’s standard genetic distance; F_st_, mean fixation index; Tajima’s D, Tajima’s population genetic test statistic.

The second to fifth column is the mean of the genetic parameter for the group. The last five columns refer to the composition of each group. When grouped by origin it is the percentage of population types, **B**reeding population, **C**ultivar, **D**iploid, **G**raminor, **L**andrace, **T**etraploid, **U**nknown and **W**ild population. When grouped by type the columns refer to the percentage of **De**nmark, **F**inland, **G**raminor, **La**ntmännen, **N**orway, **S**weden, **DL**F, **Lo**cal population or **R**ussian federation.

**Figure 2:**
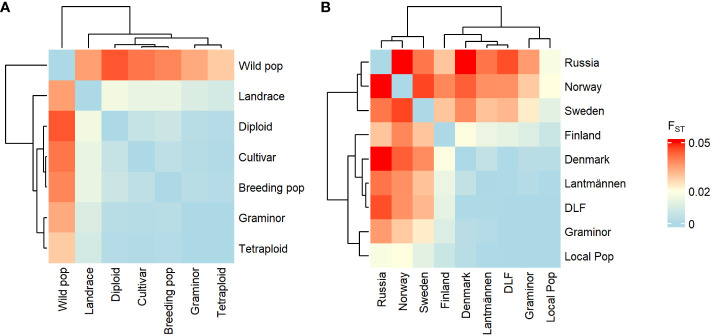
Heatmap depicting the pairwise F_ST_ values between groups of red clover populations based on population type **(A)** or origin **(B)**.

The pairwise F_ST_ between groups showed high genetic similarity between the cultivated types (**Figure 2A**) while the wild population group was divergent from the rest. Among the origin-based groups, Sweden, Norway, and Russia (which are dominated by wild populations) showed significant genetic differentiation from the other origin-based groups (forming a separate cluster in **Figure 2B**). This suggests a significant difference in allelic states between accessions from these countries and those from the other origins. Further, cultivated types as well as origin-based groups that are largely composed of cultivated types did not appear to have a clear population structure within or between them.

**Figure 3:**
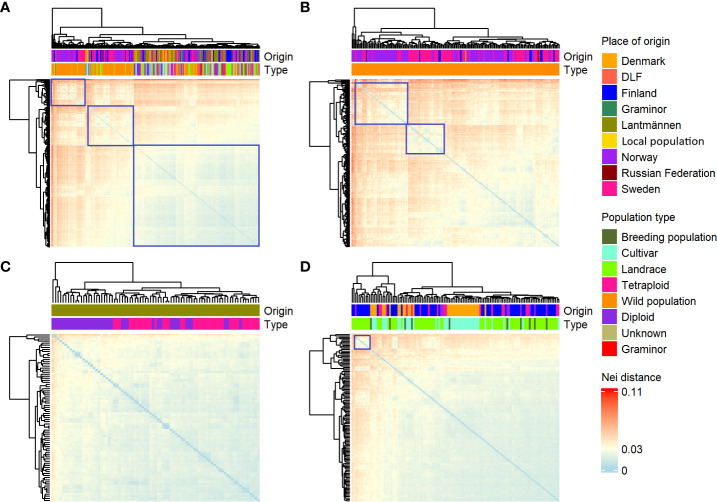
A heatmap depicting Nei’s standard genetic distances between each pair of populations. The colors indicate high (red), intermediate (yellow) or low (blue) genetic distances. The accessions were clustered according to their pairwise genetic similarities. The accessions included were **(A)** all 382; **(B)** WildOnly those that are wild; **(C)** PopulationsOnly those from Lantmännen; and **(D)** Cultivarsonly cultivars and landraces from NordGen.

The mean values of Nei’s standard genetic distance within the different groups were quite similar (**Table 1**). Hence, it was further investigated at a population level to illustrate groups of populations with high genetic similarity and had similar genetic relationships with the remaining populations. Only a few groups could be identified in the present study (marked by the blue rectangles in **Figures 3A–C**). There was a clear separation between clusters containing populations with a relatively high genetic distance (wild populations) and populations with low genetic distance (cultivars, **Figure 3A**). When the wild populations were separately analyzed, two clusters were identified, where one contained mainly the Swedish and Norwegian populations while the other contained mostly Swedish but also Finnish and Norwegian populations (**Figure 3B**). The separate analyses of the Lantmännen accessions representing the cultivated gene pool revealed similarly low genetic distance between the accessions, and no clearly defined clusters were found (**Figure 3C**). Similar results were obtained with the cultivated accessions of NordGen, with the exception of a small cluster formed by the Finish landrace accessions (**Figure 3D**).

### Cluster analysis via PCoA, DAPC and neighbor joining tree

**Figure 4:**
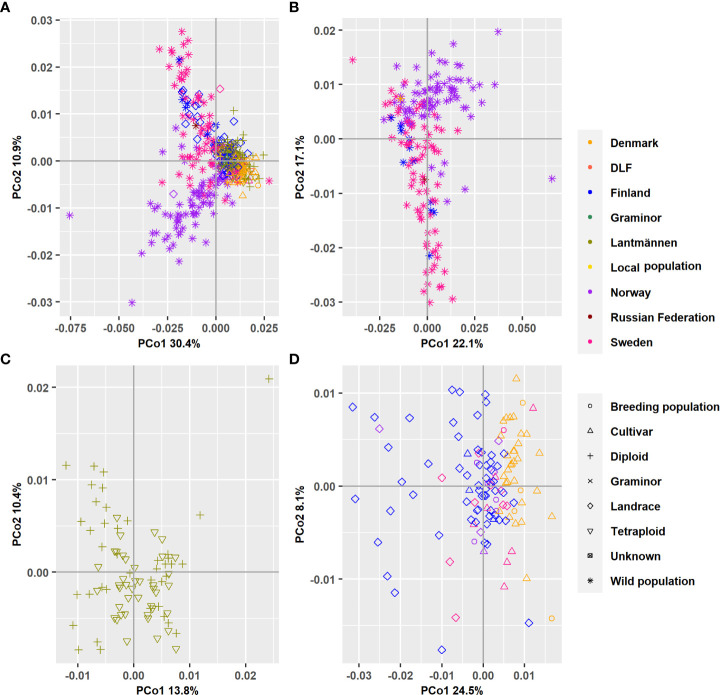
A bi-plot of the principal coordinate analysis (PCoA) showing the variation explained by the first two principal components. **(A)** All 382 populations’ analyzed together and separate analysis when the accessions have been grouped according to wild populations **(B)** Lantmännen populations **(C)** or Landraces and cultivars held at NordGen **(D)**.

A principal coordinate analysis conducted based on Nei’s standard genetic distance showed that the first principal coordinate (PCo1), which accounted for 30.8% of the total variation distinguished most of the wild populations from the cultivated ones (**Figure 4A**). It was also shown that the landrace populations were placed between the wild and cultivated populations along the PCo1. The second principal coordinate (PCo2), which accounted for 10.9% of the total variation, distinguished wild populations and one landrace population originating from Norway from a group containing wild populations from Sweden, Finland, and Russia, as well as landrace populations from Finland. The results clearly showed that the major contributors to the variation displayed in the first two principal coordinates are wild populations. Thus, to get a better understanding of the main clusters, the 382 accessions were divided into subsets. In the wild population subset, the pattern persisted as expected and the cumulative variance described by the first two PCos decreased only slightly (from 41.7% to 39.1%; **Figure 4B**).

When the Lantmännen accessions were separately analyzed, the PCoA showed a cumulative variance of 23.9% in the first two PCos (**Figure 4C**). However, the scatter plot showed no clear partition between diploid and tetraploid accessions in both the PCo1 and PCo2. The separate PCoA of the NordGen accessions revealed a major separation of the landrace accessions from cultivars and breeding populations along the first PCo, which explained 24.8% of the total variation. Furthermore, it showed a separation of populations from Denmark and Finland (**Figure 4D**). The second PCo described far less variation (8.3%) and did not show a clear separation between any of the different groups.

**Figure 5:**
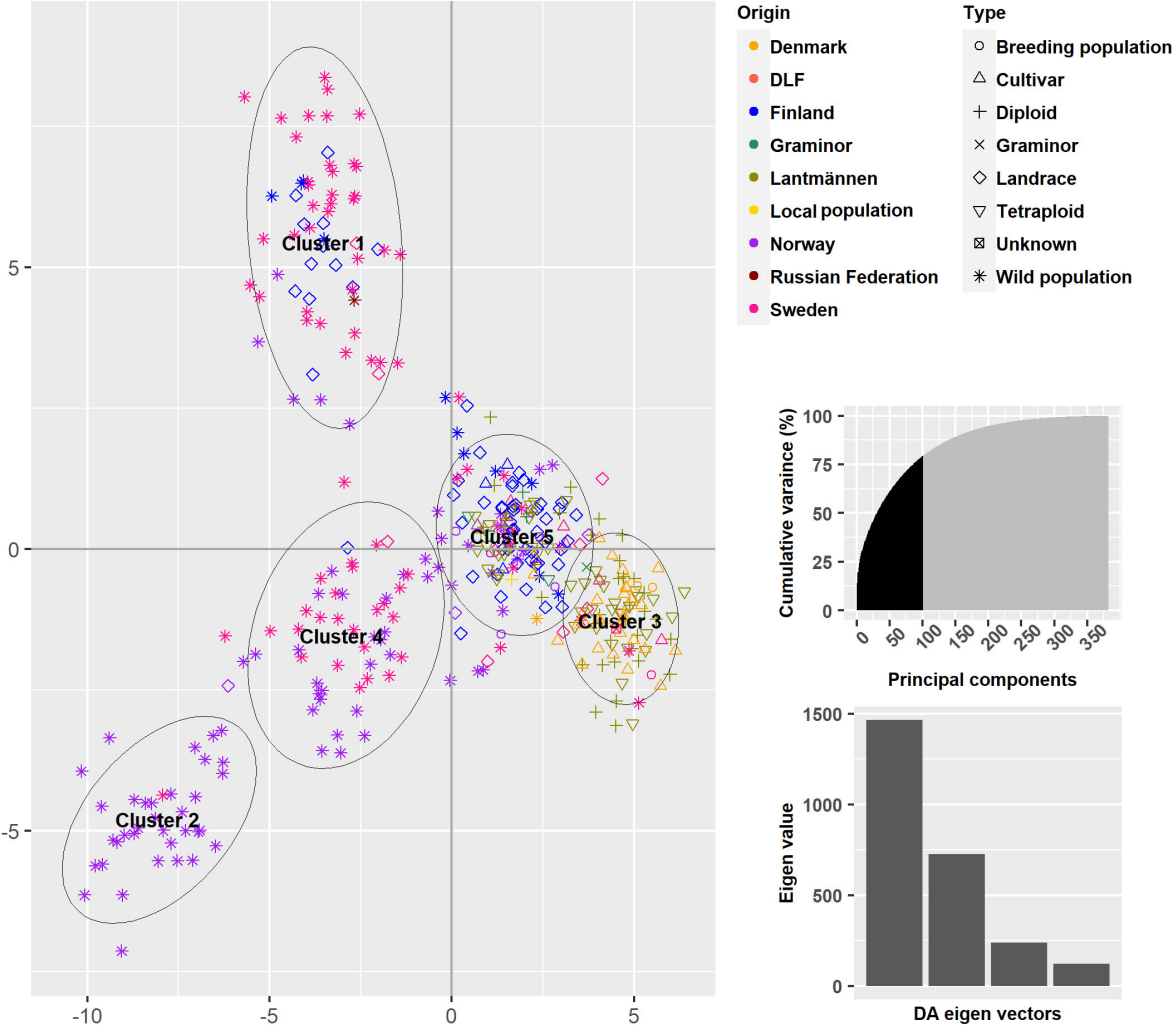
A Discriminant Analysis of Principal comonents using 150 Principal components and a five cluster solution.

A DAPC on the 382 accessions explained 79.2% of the total variance and revealed five clusters (**Figure 5** and **Supplementary Table S1**). Clusters 3 and 5 mainly comprised the cultivated types as well as some wild accessions. The major source of variation for the differentiation between clusters 3 and 5 appears to be the accessions’ countries of origin, especially Denmark versus Finland. Clusters 1, 2 and 4 differentiated the wild populations from the cultivated types although some landraces were contained in Clusters 1 and 4. Clusters 2 and 4 are dominated by wild populations from Sweden and Norway, respectively, while Cluster 1 comprised of wild populations and landraces from Finland and Sweden. This clustering follows the map Nordic Region of Europe from east to west.

**Figure 6:**
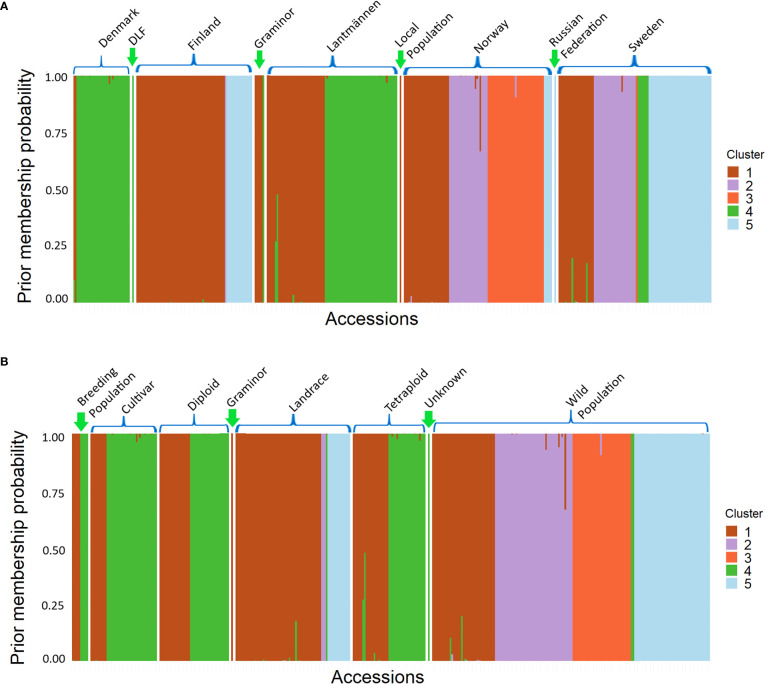
A Structure-like membership probability graph showing individual populations of different groups, classified according to **(A)** their origin and **(B)** their population type, assigned to a cluster.

Each accession has been assigned to a cluster based on a membership probability, which can be plotted in the same way as the commonly used software STRUCTURE Following the instructions provided in Jombart and Collins (2015) (**Figure 6**). The membership probabilities were high with some overlaps between cluster 1 and 2, 1 and 3 as well as 1 and 4. Here, the differentiation between Finnish and Danish populations in clusters 1 and 4 is more prominent. It is again shown by the membership of wild populations in all clusters, that the wild populations contain a high genetic variance.

**Figure 7:**
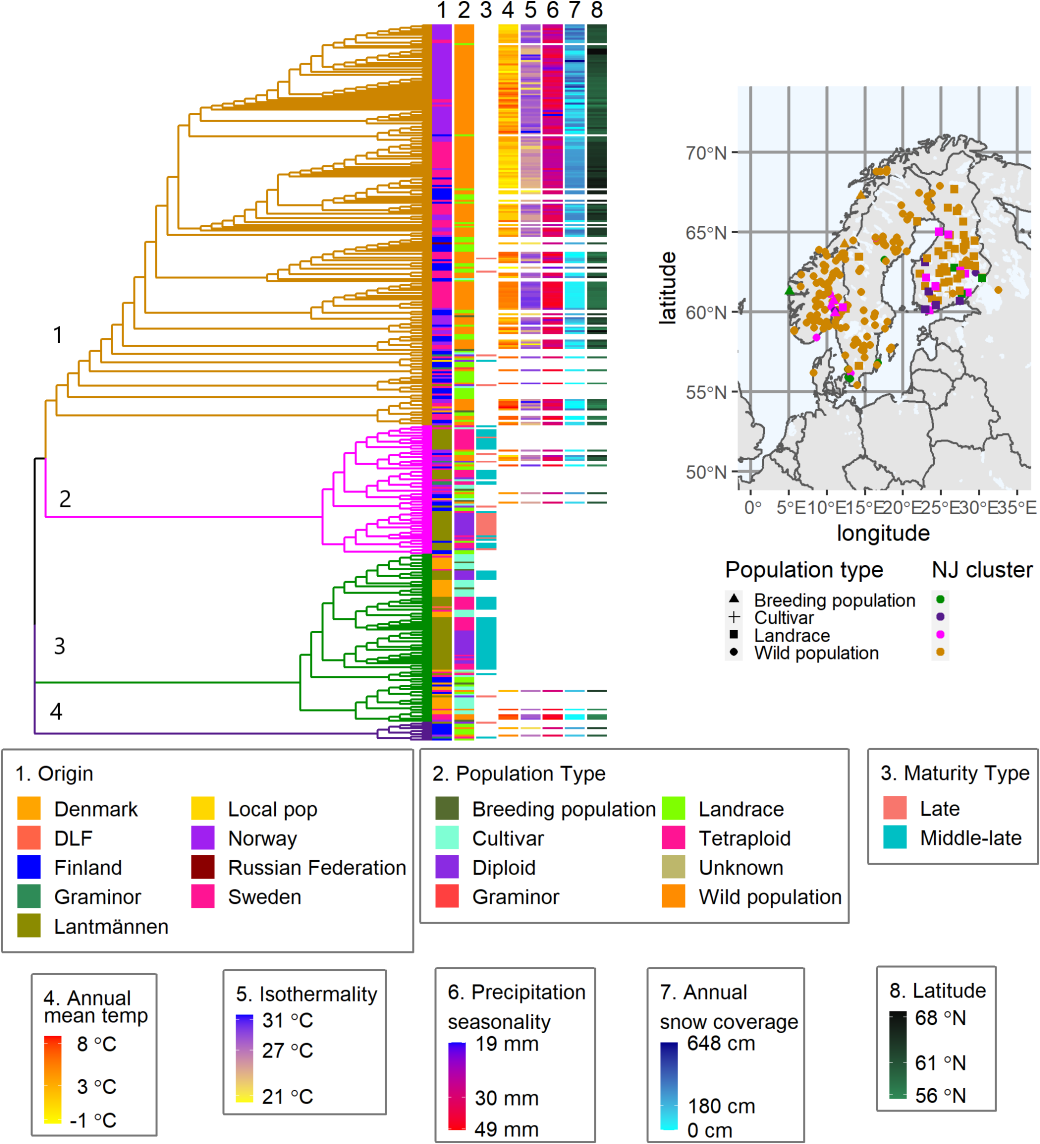
A Nei’s standard genetic distance based neighbor-joining tree of the 382 populations showing four major clusters. Each column number (1-8) links the column to a specific trait or descriptive data, 1-2 origin and type, 3 maturity types on previously scored populations. Column 4-8 is the bioclimatic variables describing the collection sites of each wild population. The geographical map shows the collection site coordinates for breeding populations, cultivars, landraces, and wild populations obtained from NordGen.

The differentiation between the cultivated and wild populations was again observed in a neighbor-joining cluster analysis based on Nei’s standard genetic distance where the 382 accessions formed four major clusters (**Figure 7** and **Supplementary Table S1**). Cluster-1 contained the majority of the wild populations, some cultivated types from both NordGen and the breeding companies. The NordGen cultivated types comprised three breeding populations from Norway, Denmark and Finland and three cultivars from Finland and Sweden. Whereas the Lantmännen cultivated types include one Graminor population and three diploid cultivars from Lantmännen. Cluster-2 and cluster-3 contained the majority of the Lantmännen accessions. Interestingly, cluster-4 contained almost exclusively Finnish accessions with the exception of one diploid cultivar and tetraploid cultivar from Lantmännen and one Norwegian wild population. Wild populations and landraces in cluster-2, cluster-3 and cluster-4 originated from along the coast or near a lake in southern to central Scandinavia. The Mantel test, which compared geographical distances with Nei’s genetic distance, revealed that isolation by distance is evident (**Supplementary Figure 1**), indicating that environmental variance could be linked to genetic variation.

The bioclimatic data from WorldClim successfully described the local environment at each of the wild populations’ sampling sites, demonstrating the variation of climate factors in the region. The five bioclimatic variables, shown in columns 4 to 8 in the heatmap of **Figure 7**, describe the average environment for each sampling site of the wild populations. They show, for example, a connection between lower annual mean temperatures and higher mean snow coverage. The highest snow coverage was recorded in the most northern geographical locations. Additionally, there were several geographical locations with high annual mean temperatures as well as high snow coverage. Similarly, there were multiple sites with high isothermality, i.e. a large difference in day to night temperatures between summer and winter. Wild red clover from these locations would have developed resilience to the harsh winter conditions, to which cultivated red clover is highly susceptible.

Interestingly, despite the fact that the wild populations with close genetic relationship to Lantmännen material do not span the entire geographical area of interest, they still represent most of the climatic conditions. Nevertheless, the main climatic conditions associated with the Lantmännen breeding materials were warmer, steady temperatures and low snow coverage. Additionally, populations with similar maturation periods clustered together in cluster-2 and cluster-3. All populations in cluster-4 with a known maturation period, except one, were late maturing. Contrary to the hypothesis, that late maturing would have larger similarities with northern wild populations, nevertheless there was no clear distinction between the maturity groups.

### LASSO models and GO analysis

In the present study, the parameter selection feature of LASSO models was used to estimate the SNPs that were the most informative in predicting the values of bioclimatic variables. A model with a root mean square error (RMSE) smaller than the standard deviation (SD) indicates that there is a predictive effect of the feature (SNP). In other words, there is an effect of the selected markers on the predictive ability of the model. All LASSO models had RMSE smaller or about equal to their respective SD (**Table 2**). Thus, all models had good predictive ability except for mean snow coverage and isothermality. The goodness of fit of the model was further confirmed by conducting a simple linear regression analysis without using the SNP information and comparing the RMSE. The RMSE of the linear regression was closer to the SD than the RMSE of the LASSO for all models except isothermality and snow coverage, thereby confirming the effect of the SNPs in the model’s prediction.

**Table 2:** A summary of the results of best preforming least absolute shrinkage and selection operator (LASSO) model and gene ontology (GO) functional enrichment analysis for the most significant single nucleotide polymorphisms (SNPs).

Environmental parameter	SD	LAMBDA	MAE	RMSE (L)	RMSE (LM)	GO	#	Go genes
Annual mean temperature	2.4	0.2	1.5	1.8	2.4	MF: kinase binding	40	8.3%
Annual precipitation	226	3.7	127	175	227	BP: stomatal opening	88	4.2%
Isothermality	2.3	0.1	1.6	2.2	2.3	None	64	
Latitude	3.1	0.2	1.7	2.1	3	None	63	
Precipitationseasonality	6.1	0.4	4.1	5.3	6.1	None	41	
Annual snow coverage	132	2.5	90	133	131	None	45	
Temperature annual range	4.4	0.1	2.3	3.0	4.4	MF: protein bindingBP: stomatal opening	74	50.8%, 4.9%

SD, standard deviation of the input data for each set of bioclimatic variables; RMSE (L), root mean square error of the LASSO model; RMSE (LM), root mean square error of a linear regression; MAE, mean absolute error of the model; GO, gene ontology; #, total number of genes; GO genes, percentage of genes that showed enrichment for each result of LASSO model.

The gene ontology (GO) enrichment analysis was used to validate the model results, in terms of biological functions. The reference gene-coding sequences, of the SNPs selected by the LASSO models were imported into the online tool Plaza workbench via BALST to find the corresponding genes. The models in which SNPs generated a GO enrichment were annual mean temperature, annual precipitation, and annual temperature range (**Supplementary Table S3**). Interestingly, the set of genes from both the annual precipitation and annual temperature models showed enrichment for genes regulating stomatal opening (**Table 2** and **Supplementary Table S3**). The stomata are known to be involved in the plants regulation of water, oxygen and carbon dioxide, functions that are relevant to changes in temperature and precipitation (Waggoner and Zelitch, 1965; Honour et al., 1995). Furthermore, there was an enrichment of genes coding for kinase binding proteins in the annual mean temperature model (**Table 2**; **Supplementary Table S3**). Kinases are a group of enzymes that via post-translational modification plays an important role in plant growth and development. Some kinases are involved in the plant response to changes in both mild and extreme temperatures (Praat et al., 2021). Analogs of the three genes detected in the GO enrichment analysis were, via experimental evidence, connected to heat response (Larkindale et al., 2005; Wu et al., 2014) and to post translational modifications as response to external factors (Colby et al., 2006) in tomato and *Arabidopsis*.

### The GO analysis of SNPs from PCA

From the PCA analysis, using the R software’s pcadapt package, the SNPs that significantly contributed to the clustering of the 382 accessions in the first two PCs were located within the coding regions of 22 and 38 genes, respectively (**Supplementary Table S3**). A GO enrichment analysis of the 22 genes from PC1 revealed that 10.5% of the genes were enriched for two biological processes, namely, specification of plant organ axis polarity and regulation of root morphogenesis. However, no GO enrichment was observed for the 38 genes from PC2.

## Discussion

This study revealed the genetic variation of 382 red clover accessions, including wild and cultivated types representing the red clover gene pool in the Nordic Region of Europe. Among the red clover accessions studied, 45 were known to be tetraploids. However, in order to facilitate the comparison with the diploid populations used in this study they were treated as diploids, following the explanation provided in Osterman et al. (2021). Diploidizing tetraploids is commonly employed to reduce complexity in data analysis and has been implemented in potato for population structure analysis using STRUCTURE and other software developed for diploids (Hirsch et al., 2013; Pandey et al., 2021; Selga, 2022). The genotyping was conducted using a pool-seq approach of the SeqSNP sequencing assay used by Osterman et al. (2021) that targeted genes known to be involved in growth and development as well as stress response. In total, 661 polymorphic, bi-allelic SNPs were detected in the targeted protein coding sequences across the 382 populations, demonstrating the potential of SeqSNP for sequencing the target SNPs as well as for *de novo* SNP discovery. Nevertheless, it should be noted that the novel SNPs identified were within 75 bp of the target SNPs. Therefore, SeqSNP is useful when specific coding regions are targeted, but may not be suitable for the identification of novel SNPs in larger regions, such as quantitative trait loci (QTL) and uncharacterized gene sequences.

The benefit of using pool-seq methods as opposed to individual sequencing methods is the number of populations that can be analyzed. With a pool of 10 individuals, pool-seq can analyze 10 times as many populations as individual genotype analysis for the same cost, assuming their sequencing depth is the same. The main challenge of pool-seq is the data analysis, as the representation of the sampled individuals within a pool can be uneven. The selection of reading depth relative to the number of individuals in each pool is very important. In the present study, using read counts from ten individuals at a sequencing depth of x501 was deemed appropriate, given the result of our in-house analysis that compared data generated through pool-seq and individual genotype sequencing.

The genetic parameters estimated from ten individuals per pool were comparable with the results reported by Jones et al. (2020) and Osterman et al. (2021). Various bioinformatics software packages have been developed to analyze pool-seq data, including BayPass (Gautier, 2015), PoPoolation (Kofler et al., 2011a; Kofler et al., 2011b), and SelEsim (Vitalis et al., 2014). Pool-seq based study on red clover using BayPass has previously been done to detect genomic signatures of herbicide resistance (Benevenuto et al., 2019). The study used pools of 20 to 40 individuals from 10 populations and data analysis was performed using BayPass, which uses read counts and a hierarchal Bayesian model to estimate genetic variance/covariance and outlier loci. However, in the present study, the number of populations (382) relative to the number of SNPs used was too large to apply BayPass.

The discovered genetic variation differentiated the groups of accessions to different extents based on their population type or origin. The major trend was a separation of the wild populations from the cultivars, with the landraces being represented within both groups. The genetic distance between the cultivated populations was low, and there was no clear separation between them based on their origins. This could be partly due to the strict outcrossing nature of the crop that facilitated a high rate of gene flow, resulting in high heterozygosity with reduced differences in allele frequency between the populations. A high level of heterozygosity has been previously reported in red clover populations analyzed at individual genotypes level, which was attributed to its strict outcrossing reproductive system (Jones et al., 2020; Osterman et al., 2021). Contrary to this, there was a pattern of population structure between the different groups of wild populations. The lower rate of gene flow between the wild populations is probably due to the low level of migration as well as the consequences of geographical distance and terrain.

This study was designed to identify informative SNPs from the perspective of red clover breeding and to generate knowledge regarding the extent to which the genetic material used for breeding reflects available genetic resources in red clover. These objectives are clearly reflected in the NJ tree (**Figure 7**) as well as in the results of LASSO model-based analysis where genetic variation was linked to bioclimatic variables and to relevant biological functions.

### Genetic Parameters: H_S_, F_ST_, Nei’s standard genetic distance and Tajima’s D

The wild populations selected for this study fully spanned the Nordic Region of Europe (**Figure 7**) with no obvious geographical groupings. The higher F_ST_ (mean of 0.04, **Table 1** and **Figure 1B**) values of the wild populations, compared to the cultivated types, suggests population structure as a consequence of either restricted gene flow, ongoing evolution, or both. In contrast, the cultivated red clover showed low F_ST_ values both within and between different cultivated types and the origins where these groups dominated (**Table 1** and **Figures 1B**, **2A**). This indicates a high gene flow within the different types of both the same and different origins. If the F_ST_ between a pair of populations is high, it implies significant differentiation between them. This means that their genetic constitution is significantly different, and hence their crossbreeding may lead to hybrids that are superior to both of them. Since low values of F_ST_ was recorded within cultivated types, crossbreeding with wild populations could lead to further genetic gain.

A lack of genetic differentiation between populations might lead to little to no genetic gain when crossbreeding. This is because the populations possess the same alleles in similar proportions across a majority of loci, and hence crossbreeding does not lead to significant genetic recombination. Even though red clover populations are expected to be highly heterozygous due to their outcrossing nature, variation between populations declines as the majority of their common alleles approach fixation. Tajima’s D can be used to measure the amount of rare alleles in a population. Hence, maintaining genetic gain in breeding populations is dependent on the inclusion of new rare alleles. Populations with negative Tajima’s D values can be considered to be in expansion following either a bottleneck or selective sweep and thus has an abundance of rare alleles (Tajima, 1989). By selecting such populations further genetic gain can be introduced into the breeding populations. The Tajima’s D for the wild populations was negative, which supports the suggestion that an ongoing evolutionary process contributes to their higher genetic differentiation. Hence, the red clover wild populations might have experienced recent selective sweeps and/or events that reduced their population sizes. Interestingly, the cultivars and breeding populations from NordGen had lower Tajima’s D mean values than the wild populations (**Table 1**). The lowest Tajima’s D values belonged to the Graminor accessions (both as origin and population type, **Table 1**). This might be due to balancing selection after the development of new cultivars or cultivar types.

Compared to the mean F_ST_ value presented by Jones et al. (2020) of 0.076 for red clover representing Europe and Asia, the mean F_ST_ of the present study (0.022) indicates lower genetic differentiation in Northern European red clover. These results are not surprising since Europe and Asia cover a larger area. Furthermore, red clover was introduced to northern Europe relatively late compared to southern and central Europe (Taylor and Quesenberry, 1996a). Additionally, Jones et al. (2020) used 93.3% ecotypes (here referred to as wild populations) compared to the 45% used in the present study. The results showed that the majority of the genetic diversity was held within the wild populations. Hence, a larger amount of wild populations would increase the F_ST_ in a sample set.

The lowest H_S_ median was recorded in the wild populations, which also had a higher genetic distance between populations compared to the other population types. The lower Hs values of the wild populations compared to the cultivated types indicate a low gene flow. Of the 382 populations selected for this study, 172 were wild populations. The large proportion of wild populations could be the reason for the relatively lower mean H_S_ of 0.18 in the present study (**Supplementary Table 1**) as compared to Osterman et al. (2021) who reported a mean H_S_ value of 0.21. Interestingly Jones et al. (2020) reported a mean H_S_ of 0.26. Therefore, the present study may suggest lower within-population diversity in Norther European wild red clover.

### Principal coordinate analysis, discriminant analysis of principal components and neighbor-joining cluster analysis

The PCoA scatter plot for the 382 populations showed a separation between cultivated and wild populations on the first principal coordinate (**Figure 3A**). The second principal coordinate differentiated Swedish and Finnish wild populations from Norwegian wild populations. The landraces from Sweden and Norway clustered together with NordGen and Lantmännen cultivars while Finnish landrace populations were closer to the Swedish and Finnish wild populations. This pattern was also observed in the DAPC, where wild populations and landraces that were not in clusters 1 and 4 exhibited three major trends, Swedish and Finnish, Swedish and Norwegian, and only Norwegian (**Figure 5** and **Supplementary Table S1**). These two main patterns were observed throughout the analysis, the separation of the Swedish wild populations from the Norwegian wild populations and the mixture of the NordGen and Lantmännen cultivars. This pattern was clearly observed in the NJ analysis (**Figure 7** and **Supplementary Table S1**) as well as in Nei’s heatmap (**Figure 2**). This distinction between cultivated and wild red clover suggests that wild populations possess genetic variation that is not represented in the cultivated populations. Hence, by incorporating wild (Norwegian or Swedish) and landrace (Finnish) populations into the breeding programs for red clover, the genetic diversity of the cultivated gene pool can be increased further.

In agreement with the results in Osterman et al. (2021), higher genetic variation differentiated wild populations from Sweden and Norway than the genetic variation that differentiated NordGen and Lantmännen cultivars or diploids and tetraploids. In order to determine the genetic relationship between populations within different groups, various analyses were conducted by grouping the 382 populations according to their origins or types, to reveal any sub-groupings. A separate PCoA analysis for the Lantmännen populations showed no clear differentiation between diploids and tetraploids, in contrast to the low degree of differentiation observed among the NordGen cultivars and landrace populations (**Figures 3C, D**). Additionally, the lack of clear differentiation between the diploids and tetraploids was evident in the DAPC, where the diploids and tetraploids were assigned to clusters 1 and 2 similarly. However, genetic variation was higher within the diploid group than within the tetraploid group. This can be seen from the PCoA scatter plot where tetraploids were distributed close to the origin while diploids covered the full range of variance described by both PCo1 and PCo2.

In agreement with the results of the PCoA and DAPC, no clear differentiation between diploids and tetraploids was described by Nei’s standard genetic distance (**Figure 2**). However, some sub-clustering of diploids and tetraploids was observed in cluster-2 and cluster-3 of the NJ tree although it was not as clear as their clustering pattern observed in Osterman et al. (2021). The grouping of the tetraploids into different sub-clusters in the present study indicates that they have been derived from chromosome-doubling experiments performed independently on diploids from different genetic backgrounds. Thus, crossbreeding of tetraploids representing different sub-clusters may result in superior cultivars with multiple desirable characteristics. Tetraploid cultivars have higher resilience and biomass yields than diploid cultivars. Hence, genetic differentiation between diploids and tetraploids is expected although it was not the case in the present study. It is likely that the agricultural gain from cultivating tetraploids derives from the molecular genetics of polyploidy rather than from an increased genetic variation since there is no clear genetic variation separating cultivars based on ploidy.

In the case of NordGen germplasm, it is interesting to note that genetic variation was higher among landraces than among cultivars, as clearly depicted in **Figure 2D**. The two groups also showed significant genetic differentiation, particularly when comparing the landraces from Finland and the cultivars from Denmark (**Figures 2D**, **5**). The distinctness of some Finnish landrace populations was also demonstrated in the heatmap of Nei’s genetic distance (**Figure 3D**) as well as in cluster-4 in the NJ tree (**Figure 7**). Hence, these Finnish landrace populations might have unique genetic constitution of significant breeding values (agronomic and forage quality) that needs to be explored further. Another interesting finding of the present study was the close genetic relationship between the Danish cultivars from NordGen and the Lantmännen populations (**Figures 2**, **4**). Possibly, this is due to the frequent inclusion of Danish cultivars in Lantmännen breeding programs or to the use of similar genetic resources by different breeding programs to develop cultivars that share similar desirable traits, such as high forage yield.

In order to understand the genetic merit of the gene pool of wild populations, the bioclimatic variables of their respective collection sites were analyzed as a means of examining their respective environments. Wild populations, even naturalized cultivars, are thought to be well adapted to the climate of their natural habitats (Turesson, 1925). Hence, a high genetic similarity between a cultivar and a wild population may indicate that the cultivar is well suited to an environment similar to that of the wild population. Such analyses can provide insight into whether the germplasm under cultivation has sufficient genetic diversity to suit the diverse environments in which they are being cultivated. The present study revealed that cultivated red clover showed a greater tendency to cluster together with wild populations found in the warmer climates of the south and central parts of the Nordic Region with low levels of variation in precipitation and temperature and little to no snow cover. One of the main causes of red clover senescence is repeated freezing and thawing (Smith, 1957; Zanotto et al., 2021). However, such information is difficult to model. Instead, an educated guess can be made using isothermality and snow coverage to identify locations that may have long autumns with frequent fluctuations around the freezing point. Wild populations from northern Norway in cluster 2 (**Figure 7**), where the snow coverage is high and the annual mean temperature is around zero or negative, shared a close genetic relationship with three middle-late diploid cultivars bred by Lantmännen. Hence, it would be interesting to evaluate the winter hardiness of these cultivars to validate the ideas discussed above.

### LASSO prediction and GO functional analysis

This study used least absolute shrinkage and selection operator (LASSO) models to relate the SNP frequency across populations to a specific bioclimatic variable. Due to its ability to rank the importance of variables LASSO models are currently used in multiple fields where the number of samples is less than the number of variables. They are used in gene-based diagnostics (Kohannim et al., 2012; Kim et al., 2018), genome-wide association research (Li et al., 2011), and other forms of unsupervised learning like in chemometrics (Pomareda et al., 2010). In the present study, the objective was to identify highly descriptive markers that can help select suitable germplasm for use in breeding programs. To the best of our knowledge, the LASSO models have not been used to relate a SNP marker to an environmental variable before. In this study, the method was considered successful because it was possible to assess the relationship between the SNPs identified by the LASSO models and the traits appropriate to the bioclimatic variable studied. Hence, this study demonstrates the ability of penalized linear regression models to assess the relationship between SNPs and environments.

Relating SNPs to bioclimatic variables with allele frequencies have previously been done via Bayesian models. However, for these models to converge, the data must fulfill the assumptions of the prior likelihood distribution. If the data does not fit the Bayesian model, a LASSO model could be used as an alternative as they rely on no prior information. LASSO models, however, are not adjusted based on population structure, unlike Bayesian models. As a result, additional steps are necessary in order to exclude possible artifacts due to population structure or statistical false discovery. A GO enrichment analysis was carried out for this purpose in the present study and satisfactory results were obtained.

Four LASSO models showed enrichment for biological processes that can be regarded as plausible responses of plants that are growing in a particular environment (**Table 2**). For example, the temperature and precipitation models showed enrichment of genes related to stomatal opening, a function known to be involved in the plant response to humidity and temperature (Waggoner and Zelitch, 1965; Honour et al., 1995). Additionally, the enrichment of protein kinases in the annual mean temperature model gives further information on the mechanisms of plant resilience. For a better understanding of persistence in red clover, a study of the stomatal changes and kinase activities in plants bearing different alleles related to their survival would be valuable. Similarly, the knowledge of how wild red clover copes with temperature stress is valuable for breeding since cold resilience is a desirable trait in Nordic breeding programs and beyond. Additionally, a study on root development between cultivated and wild red clover would be of interest given the results of the GO enrichment of auxiliary root development. The establishment of roots could affect persistence, resilience, nitrogen fixation, and nutrient uptake, as well as the establishment of the whole plant. This way of evaluating new germplasm could serve as a key component of red clover improvement.

## Conclusion

This study thoroughly described the genetic diversity and population structure of the Nordic red clover genetic resources, which include breeding populations, cultivars, landraces, and wild populations. As shown by this study, further genetic gains are possible by incorporating NordGen cultivars and landraces. Inclusion of selected landraces and wild populations based on the results exhibited in **Figure 7** into red clover breeding programs could increase persistence and climate resilience of cultivars and synthetic populations. Furthermore, GO enrichment analysis facilitated the identification of SNPs that may affect the stomatal function and root development in wild populations, thus providing additional knowledge for breeding this forage crop. It would be very interesting to see this method applied in other similar studies involving wild germplasm.

## Data availability statement

The datasets presented in this study can be found in online repositories. The names of the repository/repositories and accession number(s) can be found below: https://www.ncbi.nlm.nih.gov/; PRJNA765476.

## Author contributions

MG secured the funding with help from RO, CH, and other project participants. With inputs from CH and RO, MG designed the genotyping part of the study while JO planned the statistical analyses regarding bioclimatic variables. JO, CH, and MG conducted the greenhouse work, including sampling leaf tissue for DNA extraction. JO wrote the manuscript draft and revised it. MG, CH, and RO reviewed the different versions of the draft and assisted in the revision process. All authors contributed to the article and approved the submitted version.

## Funding

The study was fully funded by SLU Grogrund – Centre for Breeding of Food Crops, Swedish University of Agricultural Sciences.

## Acknowledgments

We would like to thank Linda Öhlund (Lantmännen) for providing us with seeds of red clover cultivars and breeding populations. We would also like to thank NordGen for supplying us with selected red clover germplasm from their collection. We are thankful to Mohammad El-Khalifeh (NordGen) for the additional help in selecting the accessions, and to the greenhouse management staff for keeping the plants healthy. We would like to thank David Parsons (SLU), Elisabet Nadeau (SLU) and Alf Ceplitis (Lantmännen) for constructive comments on the results of the study during the project meetings.

## Conflict of interest

The authors declare that the research was conducted in the absence of any commercial or financial relationships that could be construed as a potential conflict of interest.

## Publisher’s note

All claims expressed in this article are solely those of the authors and do not necessarily represent those of their affiliated organizations, or those of the publisher, the editors and the reviewers. Any product that may be evaluated in this article, or claim that may be made by its manufacturer, is not guaranteed or endorsed by the publisher.
