# Supplementary figures and images for: Discovering candidate SNPs for resilience breeding of red clover
Source: Front Plant Sci. 2022 Sep 28;13:997860. doi: 10.3389/fpls.2022.997860 (PMC9554550; doi:10.3389/fpls.2022.997860)

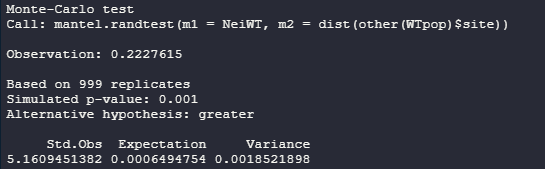

Supplement: Supplementary file 1 [file Image_1.png]
